# Clinical outcomes of rib fracture stabilization and conservative treatment in a high-volume Asian trauma center: a propensity score-matched retrospective study

**DOI:** 10.1186/s13017-025-00620-8

**Published:** 2025-05-19

**Authors:** Chia-Cheng Kao, Ke-Cheng Chen, Xu-Heng Chiang, Jen-Hao Chuang, Chao-Wen Lu, Wei-Ling Hsiao, Tzu-Hsin Lin, Hsien-Chi Liao

**Affiliations:** 1 Department of Surgery, National Taiwan University Hospital, Taipei, Taiwan; 2 Division of Thoracic Surgery, Department of Surgery, National Taiwan University Hospital, Taipei, Taiwan; 3 Department of Medical Education, National Taiwan University Hospital, Taipei, Taiwan; 4 Department of Surgical Oncology, National Taiwan University Cancer Center, Taipei, Taiwan; 5 School of Nursing, National Taiwan University College of Medicine, Taipei, Taiwan; 6 Department of Nursing, National Taiwan University Hospital, Taipei, Taiwan; 7 Department of Traumatology, National Taiwan University Hospital, 7 Chung-Shan South Road, Taipei, Taiwan; 8 Graduate Institute of Clinical Medicine, National Taiwan University College of Medicine, Taipei, Taiwan

**Keywords:** Rib fractures, Surgical fixation, Conservative treatment, Acute pain, Length of stay

## Abstract

**Background:**

Rib fractures are common chest wall injuries with conservative treatment and surgical stabilization of rib fractures (SSRF) as treatment options. We retrospectively compared the efficacy and long-term prognosis of conservative treatment and SSRF as treatment options for rib fractures.

**Methods:**

This retrospective study was conducted at a single trauma center in Taiwan. The study population comprised patients with rib fractures who underwent conservative treatment or SSRF at the National Taiwan University Hospital between 2017 and 2022. We analyzed the outcomes between the operative and non-operative groups, including the length of intensive care unit and hospital stays, pain scales at admission and follow-up, and post-operative complication rates.

**Results:**

Of the 217 patients with rib fractures in this study, 103 received SSRF, and 114 received conservative treatment. Patients in the operative group had worse consciousness statuses and higher injury severity scores than those in the non-operative group. In addition, patients in the operative group had more preoperative chest complications than those in the non-operative group. Regarding outcomes and long-term prognoses, patients in the operative group had longer intensive care unit and hospital stays than those in the non-operative group; however, patients in the operative group had better recovery quality than those in the non-operative group.

**Conclusions:**

Our study showed that, in patients who meet the surgical indications, SSRF is an effective and safe way to relieve acute pain after thoracic injury and achieve better recovery and quality of life after surgical intervention.

## Background

Rib fractures are common chest wall injuries usually caused by blunt force to the chest wall. Rib fractures can also be caused by progressive coughing, exercise, or non-accidental trauma [1, 2], especially in patients with osteoporosis, chronic obstructive pulmonary disease, or bronchial asthma, receiving long-term steroid therapy [2]. In addition, malignancies with bone metastases can also cause pathological rib fractures [3, 4]. Clinical manifestations of rib fractures include minimal symptoms, such as mild painful sensations in the chest and intermittent dyspnea, as well as severe life-threatening conditions, such as tension pneumothorax and massive hemothorax [1, 2, 5].

Clinically, rib fracture treatments can be divided into two strategies: conservative and surgical treatments [5–8]. If there is no indication for surgery based on the medical history, detailed physical examination, and comprehensive imaging examinations, conservative treatment is usually administered [5, 9]. Conservative treatment focuses on effective pain control and prevention of subsequent pulmonary complications, such as lung collapse or pneumonia. According to the SABRE randomized clinical trial, in addition to traditional non-steroidal anti-inflammatory drugs and opioids, serratus anterior plane blocks are also effective in reducing pain in these patients [10]. Furthermore, epidural analgesia may also play a role in reducing pain during hospitalization [11]. Oxygen therapy or intercostal tube drainage may be indicated in severe clinical situations to assist the expansion of the collapsed lung and reduce the risk of subsequent pulmonary infections, lung abscesses, or empyema.

The current mainstream surgical treatment method for rib fractures is minimally invasive surgery [6, 7]. In surgical stabilization of rib fractures (SSRF), the fractured ribs are returned to their original position and fixed with metallic plates and screws. The thoracoscopy-assisted method can also be used to drain blood clots and pleural effusions or to repair lung lacerations for pulmonary complications such as hemothorax or pneumothorax during surgery [12].

Several international studies have shown that SSRF can effectively relieve acute pain, increase the patient’s quality of life during the recovery period, and have a better effect on rib fracture healing. In patients with flail chests, a meta-analysis in 2022 showed that surgical fixation of flail sections and multiple rib fractures reduced morbidity and mortality outcomes compared to conservative treatment [6]. Another similar study also showed that surgical treatment was preferable to non-surgical management in reducing pneumonia, chest deformities, tracheostomies, mechanical ventilation durations, and lengths of intensive care unit (ICU) stays [13]. For patients with non-flail chest rib fractures, surgery also had some benefits. A prospective cohort study in China showed that minimally invasive surgery (MIS) was a simple and safe treatment that significantly relieved chest pain, rapidly restored pulmonary function, and improved the long-term quality of life of patients with non-flail chest rib fractures [14]. A previous study showed that, besides trauma-induced rib fractures, for patients with symptomatic rib nonunion and in some selected symptomatic patients, surgical fixation is safe and feasible with low perioperative complication rates [15]. Another scoping review in 2024 showed similar results, wherein surgical management of rib fracture nonunion was shown to be an effective treatment with acceptable implant failure and complication rates [16]. Therefore, surgical management is a viable option for symptomatic patients [16].

At the National Taiwan University Hospital, patients with rib fractures had four unique characteristics. First, there was a high percentage of motorcycle accident-induced multiple rib fractures caused by the high rate of daily motorcycle usage in Taiwan. Second, owing to the accessibility of computed tomography, chest computed tomography imaging, and three-dimensional reconstruction were performed before the operation to obtain a detailed examination of the rib fracture positions. Third, the patient’s pain scale upon admission and during the follow-up at the outpatient clinic was recorded. Finally, the patient’s pulmonary function before and after surgery was followed up to evaluate the surgical outcome objectively. In a previous study, we conducted a prospective cohort study of patients with multiple rib fractures admitted to the National Taiwan University Hospital between July 2017 and June 2019 to compare the postoperative outcomes and 6-month quality of life between surgical and conservative treatment.^17^ The results showed that surgical rib fixations accelerate recovery in patients with severe thoracic injuries (chest abbreviated injury scale [AIS] ≥ 3) and achieve 6-month quality of life comparable to patients with less severe injuries (chest AIS ≥ 2) [17].

Furthermore, in this study, we retrospectively collected and reviewed the data of all patients with rib fractures at the National Taiwan University Hospital between January 1st, 2017, and December 31st, 2022, and analyzed the clinical outcomes between SSRF and conservative treatment.

## Methods

### Patient demographics

This retrospective study was conducted at a single medical center. The study population comprised patients with rib fractures who received conservative treatment or SSRF at the National Taiwan University Hospital between January 1st, 2017, and December 31st, 2022. This study was reviewed and approved by the Institutional Research Ethics Committee (202408012RINC) and conducted in accordance with the Declaration of Helsinki. We reviewed the patients’ basic personal information, including age, sex, body mass index (BMI), smoking history, and underlying diseases, including asthma, chronic obstructive lung disease, hypertension, diabetes mellitus, and coronary artery diseases. In addition, we also recorded the causes of rib fractures, such as motorcycle accidents, car accidents, falls, and pedestrians hit by cars. The consciousness level and injury severity were also recorded and presented along with the Glasgow Coma Scale (GCS) and injury severity score (ISS), respectively. Rib fracture patterns were documented, including the total number of ribs fractured, rib fracture side, and other chest complications such as displacement, pleural effusion, and pneumothorax. Finally, we calculated the rib fracture score (RFS), which represented the rib fracture severity, defined as the number of ribs fractured multiplied by the rib fracture sides with age added.

### Surgical indications

The definitive indications for SSRF at the National Taiwan University Hospital included (1) a flail chest with paradoxical breathing, (2) multiple rib fractures, defined as ≥ 3 ribs fractured, combined with uncontrolled hemopneumothorax, which defined as persistent accumulation of air and blood in the pleural cavity that does not respond to standard chest tube drainage, potentially causing respiratory or hemodynamic instability and requiring surgical intervention such as SSRF, and (3) severe thoracic injury related to respiratory failure, such as desaturation, hypocapnia, and prolonged intubation, without other medical causes. If the patient fulfilled one of these three indications, SSRF was indicated. In addition to absolute surgical indications, relative surgical indications included chronic neuralgia with inadequate effect of medical pain management, symptoms or signs associated with rib nonunion, such as dyspnea and restrictive lung disease, chest wall deformity, or other cosmetic reasons, and personal factors.

### Surgical techniques

Patients with a history of trauma, including vehicle accidents or falls, or who had symptoms of dyspnea and chest pain were sent to the emergency department at National Taiwan University Hospital, where chest computed tomography was performed to evaluate the thoracic injury severity. If the patient was diagnosed with a rib fracture and met one of the three definite surgical indications mentioned above, surgical treatment was administered unless contraindications such as extremely old age, coagulopathies, and severe infections were present. During surgery, general anesthesia with endotracheal intubation was performed. An incision approximately 10 cm in length was made at the location of the rib fracture. The fractured ribs were returned to their original positions using forceps and fixed with metallic plates and screws. We then created a working port in the intercostal space and used video-assisted thoracoscopy to perform hematoma evacuation, lung laceration repair, or decortication. Following the procedure, an intercostal drain was inserted into the pleural space. A CWV^®^ drainage tube was placed above the muscle layer for subcutaneous drainage. After surgery, the patients were transferred to the ICU for further surveillance if extubation was not suitable immediately after surgery. The patient was discharged after the intercostal drainage tube was removed and followed up at the outpatient clinic of the National Taiwan University Hospital.

### Outcomes

The outcomes between the operative and non-operative groups were analyzed according to the length of ICU and hospital stays, pain scales at admission and at follow-up at the outpatient clinic (72 h after injury, 2 weeks postoperatively, and 3 months postoperatively), and complication rates.

### Statistical analyses

We analyzed the collected data using IBM SPSS Statistics Software, 27th edition. A two-sample t-test was used to analyze the differences between the operative and non-operative groups. P-values < 0.05 were considered statistically significant.

## Results

**Fig. 1:**
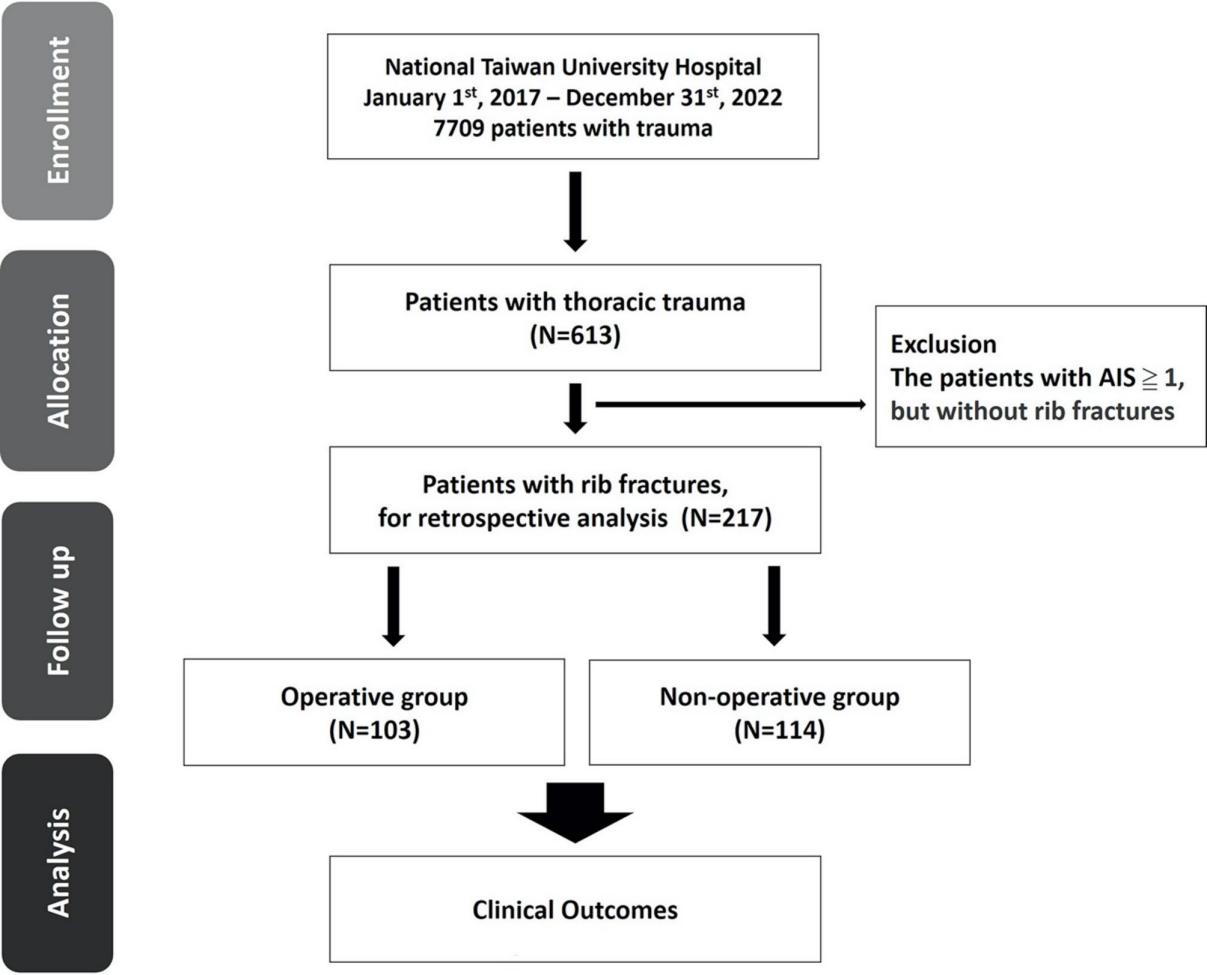
Flow diagram of the study

A total of 217 patients were included in the study. Among the included patients, 103 underwent surgical fixation, and 114 underwent conservative treatment (Fig. 1).

No statistically significant differences were observed in the basic demographics of the two groups, including age, sex, BMI, smoking history, and underlying diseases. Regarding the mechanism that caused the rib fractures, most were motorcycle accidents, which accounted for approximately 44–45%, followed by falls. However, patients in the operative group had significantly worse consciousness statuses and higher ISSs than those in the non-operative group (Table 1).

**Table 1:** Patient demographics

Variable	Operative group (*N* = 103)	Non-operative group (*N* = 114)	*P*-value
Age (years) (Mean ± SD)	57.62 ± 15.95	58.41 ± 18.073	0.077
Sex (Male/Female)	68/35	79/35	
Body mass index (kg/m^2^)	25.17 ± 4.31	24.72 ± 3.98	0.607
Smoking History			
Smoker	25 (24.3%)	26 (22.8%)	0.614
Non-smoker	78 (75.7%)	88 (77.2%)	
Underlying Diseases			
Asthma	4 (3.9%)	4 (3.5%)	0.771
COPD	4 (3.9%)	5 (4.4%)	0.712
Hypertension	46 (44.6%)	49 (43.0%)	0.626
DM	27 (26.2%)	26 (22.8%)	0.248
CAD	10 (9.7%)	13 (11.4%)	0.420
Mechanism of injury			
Motorcycle accident	46 (44.6%)	51 (44.7%)	
Car accident	3 (2.9%)	4 (3.5%)	
Fall	1 (0.9%)	1 (0.8%)	
Fall down	26 (25.2%)	43 (37.7%)	
Pedestrian hit by car	11 (10.7%)	3 (2.6%)	
Others	3 (2.9%)	12 (10.5%)	
GCS (Mean+/-SD)	14.47 ± 1.69	14.87 ± 0.45	< 0.001
ISS (Mean+/-SD)	20.54 ± 8.879	16.77 ± 8.943	0.511
Head AIS	0.76 ± 1.457	0.59 ± 1.299	0.090
Face AIS	0.10 ± 0.389	0.24 ± 0.634	< 0.001
Chest AIS	3.66 ± 0.655	3.01 ± 0.903	0.222
Abdomen AIS	0.35 ± 1.029	0.69 ± 1.287	< 0.001
Extremity AIS	1.19 ± 1.220	1.01 ± 1.150	0.087
Appearance AIS	0.02 ± 0.200	0.04 ± 0.325	0.244
Head injury AIS code ≥ 2	22 (21.3%)	15 (13.1%)	0.001

AIS, abbreviated injury scale; CAD, coronary artery disease; COPD, chronic obstructive pulmonary disease; DM, diabetes mellitus; GCS, Glasgow Coma Scale; ISS, injury severity score; SD, standard deviation.

In the operative group, a significantly higher percentage of patients received surgical interventions other than SSRF, especially clavicle open reduction and internal fixations (ORIFs). The operative group had a higher number of rib fractures and more chest complications before surgery than the non-operative group, including displacements, pleural effusions, and pneumothoraces. Moreover, patients in the operative group had higher RFSs than those in the non-operative group. Most of the fractured ribs in both groups were on the left side (Tables 2 and 3).

**Table 2:** Associated injury description

Variable	Operative group (*N* = 103)	Non-operative group (*N* = 114)	*P*-value
Other surgical intervention required			
Clavicle ORIF	30 (29.0%)	8 (7.0%)	< 0.001
Neurosurgery (Head Trauma)	5 (4.9%)	1 (0.9%)	< 0.001
Extremity	15 (14.6%)	11 (9.6%)	0.026
Others (Maxillofacial, Abdominal and Pelvic Surgery)	28 (27.2%)	13 (11.4%)	< 0.001

ORIF, open reduction and internal fixation

**Table 3:** Rib fracture patterns

Variable	Operative group (*N* = 103)	Non-operative group (*N* = 114)	*P*-value
Total number of fractured ribs (mean ± SD)	6.36 ± 2.627	4.9 ± 2.082	0.162
Ribs fracture side			
Right only	39 (37.8%)	35 (30.7%)	
Left only	61 (59.2%)	67 (58.7%)	
Bilateral	3 (2.9%)	12 (10.5%)	
Rib fracture			
1–2	2 (1.9%)	12 (10.5%)	
3–5	38 (36.9%)	63 (55.3%)	
≧ 6	63 (61.2%)	39 (34.2%)	
Displacement	60 (58.2%)	24 (21.0%)	< 0.001
Pleural effusion	99 (96.1%)	36 (31.6%)	< 0.001
Pneumothorax	47 (45.6%)	29 (25.4%)	< 0.001
Rib Fracture Score	9.214 ± 4.689	8.246 ± 4.012	0.907

SD, standard deviation

Regarding SSRFs, the mean number of days to operation at the National Taiwan University Hospital was 4.34 ± 2.97 days. The mean operation time was 152.78 ± 46.421 min. The mean rib fixation number was 3.12 ± 1.032. Post-operative complications included two subcutaneous hematomas, which accounted for 1.9% of the patients in the operative group (Table 4).

**Table 4:** Surgical analysis

Variable	Operative group (*N* = 103)	Non-operative group (*N* = 114)	*P*-value
Time to Operation (days)	4.34 ± 2.97		
Rib Fixation Numbers (mean ± SD)	3.12 ± 1,032		
Rib Operation Time (mean ± SD) (minutes)	152.78 ± 46.421		
Post-OP Pulmonary Complication (Pneumonia, Hemothorax)	2 (1.9%)		

SD, standard deviation

According to our study, patients with rib fractures in the operative group had longer ICU and hospital stays than those in the non-operative group. The intubation rate in the surgical group (34.0%) was also significantly higher than in the non-surgical group (7.9%). However, the operative group had significantly better quality of recovery during long-term follow-up and presented with a lower pain scale during outpatient clinic follow-up compared with the non-operative group, especially the pain scale 3 months post-operatively (Table 5).

**Table 5:** Clinical outcomes

Variable	Operative group (*N* = 103)	Non-operative group (*N* = 114)	*P*-value
Duration of ICU stay (days) (mean ± SD)	4.35 ± 4.76	1.52 ± 3.06	0.006
Intubation			
Yes	35 (34.0%)	9 (7.9%)	< 0.001
No	68 (66.0%)	105 (92.1%)	
Tracheostomy			
Yes	0 (0%)	0 (0%)	< 0.001
No	103 (100%)	114 (100%)	
Duration of intercostal drain insertion (days)	5.14 ± 3.337		
Duration of hospital stay (days)	12.97 ± 8.044	7.99 ± 8.635	0.762
Pain scale			
Injured − 72 h	5.19 ± 2.86	6.30 ± 2.379	0.088
Postoperative 2 weeks	2.59 ± 1.86	4.62 ± 2.27	0.056
Postoperative 3 months	1.29 ± 1.50	2.44 ± 1.90	0.021

SD, standard deviation

## Discussion

This study showed no significant differences in patient demographics, including age, sex, BMI, smoking history, and other underlying diseases, between the operative and non-operative groups. Motorcycle accidents were the primary cause of multiple rib fractures at the National Taiwan University Hospital (operative group, 44.6%; non-operative group, 44.7%), which may be due to the high rate of motorcycle use in Taiwan [18]. In Taiwan, nearly 60% of all driving fatalities involve motorcycles. The second most common cause of rib fractures was falls.

Patients in the operative group had higher head AIS scores and worse consciousness statuses with lower GCS scores, indicating that this group had clinically more severe injuries and may have needed other surgical interventions in addition to SSRFs, especially clavicle ORIFs. The clavicle is a body part that is usually injured during motorcycle accidents [19]. According to a previous systemic review, clavicle and rib fractures are closely related in patients with poly-trauma, and almost a fifth of all patients with blunt chest trauma sustain both injury types [19].

As Taiwan is a left-driving country, the primary side of rib fractures was the left side. Patients in the operative group had more thoracic complications than those in the non-operative group, including displacements, pleural effusions, and pneumothoraces, indicating that this group had a more critical clinical status. A previous study showed that rib fixation may benefit selected patients with traumatic rib fractures and much more severe clinical conditions, such as those with bilateral rib fractures, multiple displaced rib fractures, flail segments, and concomitant thoracic injuries [20].

The mean time to surgery in our hospital was 4.34 ± 2.97 days, approximately 96 h. A previous retrospective study that included 2,839 patients, of whom 1,520 (53.5%) underwent early surgical stabilization of rib fractures (< 72 h), showed that early surgical stabilization of rib fractures decreased the length of hospital and ICU stay and lowered the rates of unplanned intubations, unplanned ICU admissions, and tracheostomies [21]. In our hospital, the strategy for rib fractures was to observe initially and perform surgical management if the clinical status matched the surgical criteria. In our study, we did not conduct a subgroup analysis between the early and late operations.

In this study, patients in the operative group had longer hospital and ICU stays and higher intubation rates. This finding differs from a previous study that showed a statistical benefit of surgical fixation compared to conservative management of rib fractures in terms of ICU length of stay, mechanical ventilation, mortality, pneumonia, and tracheostomy [6]. This different result may be because of the higher proportion of patients undergoing other surgical interventions, thus extending the recovery period and length of hospital stay. However, in the operative group, the pain scale was much lower at 2 weeks and 3 months after the operation than in the non-operative group, indicating that the patients in the operative group had a significantly better quality of recovery after surgery than the non-operative group. In a previous randomized controlled trial of patients with severe chest trauma, SSRF increased hospital length of stay and did not provide any quality of life benefit for up to 6 months [22]. In our study, surgical intervention showed a better long-term prognosis for these patients.

Between 2017 and 2019, our medical institution conducted a prospective study that compared the long-term outcomes of non-surgical and surgical management of rib fractures in patients with major trauma without head injuries. And showed that surgical management of rib fractures can reduce pain and hospital stay in patients with major trauma [23]. In this study, we retrospectively collected data on all patients with rib fractures diagnosed in our hospital and showed that surgical management could reduce the pain scale and increase the quality of life, even in patients with simultaneous head injuries.

However, this study had some limitations. First, owing to this study’s retrospective design, there may have been bias in collecting and reviewing patient data. Some data may have been obscured, and some may have been lost during follow-up. Second, the pain scale is a subjective method for evaluating clinical outcomes; the lack of objective evaluation may have also introduced some bias. Third, our study did not focus on subgroup analysis or evaluation of the surgical stabilization of rib fractures in patients with non-multiple rib fractures. The final item, the lack of data on the degree of displacement of rib fractures, which may influence the decision-making process for surgical stabilization. Future studies should include this variable to better stratify patients and evaluate outcomes.

## Conclusions

Our study showed that once patients with rib fractures meet the surgical indications for SSRF, this type of surgery is an effective and safe way to relieve acute pain after thoracic injury and achieve better recovery and quality of life after surgical intervention. However, to validate these findings further, studies with sub-group analyses, especially for SSRF in patients with non-multiple rib fractures, are needed.

## Data Availability

No datasets were generated or analysed during the current study.

## Abbreviations

AIS: Abbreviation injury scale
BMI: Body mass index
GCS: Glasgow Coma Scale
ICU: Intensive care unit
ISS: Injury severity score
MIS: Minimally invasive surgery
ORIF: Open reduction and internal fixation
RFS: Rib fracture score
SSRF: Surgical stabilization of rib fractures
